# Analysis of Repeated Measurements of Serum Carotenoid Levels and All-Cause and Cause-Specific Mortality in Japan

**DOI:** 10.1001/jamanetworkopen.2021.13369

**Published:** 2021-06-11

**Authors:** Ryosuke Fujii, Yoshiki Tsuboi, Keisuke Maeda, Yuya Ishihara, Koji Suzuki

**Affiliations:** 1 Department of Preventive Medical Sciences, Fujita Health University School of Medical Sciences, Toyoake, Japan

## Abstract

**Importance:**

The associations of levels of diverse serum carotenoids ascertained via repeated measurements with all-cause, cancer, and cardiovascular disease (CVD) mortality risk have not been considered in previous prospective studies.

**Objective:**

To investigate the association between repeated measurement of serum carotenoid levels and all-cause and cause-specific mortality risk.

**Design, Setting, and Participants:**

This cohort study’s baseline data were collected using information from physical examinations from 1990 to 1999. Eligible participants were followed up until December 2017, with a median (interquartile range) follow-up period of 22.3 (15.5-25.3) years. Included individuals were age 40 years or older at the baseline data collection, were residents of the study site in the town of Yakumo, Hokkaido, Japan, and participated in a physical examination at least once from 1990 to 1999. Among eligible participants, after excluding 332 individuals, 3116 individuals were included in the analysis. Data analysis was conducted in April 2020.

**Exposures:**

Repeated measurements of 6 serum carotenoid levels and 4 associated indices.

**Main Outcomes and Measures:**

All-cause, cancer, and CVD mortality, categorized by *International Classification of Diseases, Ninth Revision* and *International Statistical Classification of Diseases and Related Health Problems, Tenth Revision* codes, were recorded. A time-dependent Cox regression model was performed to examine associations between time-varying serum carotenoid levels and mortality.

**Results:**

Among 3116 individuals who received physical examinations, the mean (SD) age was 54.7 (10.6) years and 1883 (60.4%) were women. During the follow-up period, 762 deaths from all causes, 253 deaths from cancer, and 210 deaths from CVD were ascertained. In a time-dependent Cox regression analysis, for every 25% higher serum levels of total carotenoids, risks were statistically significantly lower for all-cause mortality (hazard ratio [HR], 0.85; 95% CI, 0.82-0.87; *P* < .001), cancer mortality (HR, 0.82; 95% CI, 0.78-0.87; *P* < .001), and CVD mortality (HR, 0.86; 95% CI, 0.81-0.91; *P* < .001). Using only baseline measures, for every 25% higher serum levels of total carotenoids, risks were also statistically significantly lower for all-cause mortality (HR, 0.92; 95% CI, 0.89-0.95; *P* < .001), cancer mortality (HR, 0.87; 95% CI, 0.83-0.93; *P* < .001), and CVD mortality (HR, 0.93; 95% CI, 0.88-0.99; *P* = .03) but with larger HRs than those associated with repeated measurements.

**Conclusions and Relevance:**

This study found that higher levels of serum carotenoids in analysis using repeated measurements were associated with significantly lower all-cause and cause-specific mortality over a follow-up period of 25 years.

## Key Points

**Question:**

Are repeated measurements of serum carotenoid levels associated with all-cause and cause-specific mortality?

**Findings:**

In this cohort study of 3116 individuals who received physical examinations, higher levels of serum carotenoid ascertained with multiple measurements were associated with lower risks of all-cause, cancer, and cardiovascular disease mortality. The decreases in risk associated with higher serum carotenoid levels were greater for analysis using repeated measurements of serum carotenoid compared with analysis using single measurements.

**Meaning:**

These findings suggest that higher carotenoid levels are associated with lower risk of mortality.

## Teaser

This cohort study investigates the association of serum carotenoid levels found using repeated measurements with all-cause, cancer, and cardiovascular disease mortality in Japanese adults.

## Introduction

Dietary intake of vegetables and fruits has been found to contribute to the reduced risks of overall and cause-specific mortality.^1,2,3^ The protective effects associated with vegetables and fruits may be attributed to the carotenoids they contain, which are mainly found in green and yellow vegetables and fruits.^4^ Serum carotenoids act as antioxidants in the human body, and their association with human health has received considerable attention for several decades. Although some intervention studies have found adverse cancer outcomes associated with serum carotenoids among individuals who smoke and workers exposed to asbestos,^5,6^ numerous prospective and interventional studies have reported the association of carotenoids with prevention of cancer, cardiovascular disease (CVD), and other pathophysiological conditions.^7,8,9,10,11,12,13,14,15,16,17^

A considerable number of prospective studies^10,11,12,13^ examined the association between baseline serum carotenoid levels and all-cause, cancer, and CVD mortality. However, most studies did not consider changes in serum carotenoid levels over a long follow-up period. Given that serum carotenoid level is a better indicator associated with dietary intake of vegetables and fruits in a time series, previous studies failed to reflect changes in nutritional status during follow-up for risk estimation. Nutrient intake can be affected by multiple internal and external factors, such as physiological status with aging, food supply, and public perception.^18^ Therefore, a prospective study with repeated measurements of serum carotenoid levels may be able to capture changes in dietary vegetable and fruit intake and is essential to estimate more precise associations with mortality. Previous longitudinal studies assessed the association of repeated measurements of serum carotenoid levels with the incidence of breast cancer^19^ and colorectal cancer.^20^ In these studies, the authors applied time-dependent analyses and found evidence that several carotenoids were inversely associated with cancer incidence. Inspired by these findings for cancer incidence, we hypothesized that higher levels of serum carotenoid using repeated measurements would be associated with a greater decrease in risks of mortality compared with the results using only baseline measurements. However, to the best of our knowledge, few studies have been conducted to examine the associations between repeated measurements of serum carotenoid levels and all-cause and cause-specific (ie, cancer and CVD) mortality risk. Therefore, we aimed to examine the association between time-varying serum carotenoid levels and all-cause and cause-specific mortality risk using a time-dependent Cox regression model.

## Methods

### Study Population

This study was conducted based on an annual population-based physical examination in the town of Yakumo, Hokkaido, Japan. The graphical timeline is shown in the Figure. The baseline data were collected using information from the physical examination from 1990 to 1999. The eligibility criteria for this cohort study were being age 40 years or older at the baseline data collection, residing at the study site, and participating in the physical examination at least once from 1990 to 1999. A total of 3448 individuals were eligible, among whom 332 individuals were excluded from the study; 63 individuals were excluded owing to a lack of informed consent, 261 individuals owing to missing values for questionnaire-based clinical history (including 84 individuals missing values for stroke, 84 individuals missing values for diabetes, 195 individuals missing values for cancer, and 96 individuals missing values for angina, with overlap among these groups), and 8 individuals owing to invalid information about end points, such as date and cause of death. Finally, we included 3116 individuals (1233 [39.6%] men and 1883 women [60.4%]) in our statistical analysis. The flow of selecting eligible participants is shown in eFigure 1 in the Supplement. The eligible participants were followed up until December 2017 using mortality records with permission from the Japanese Ministry of Internal Affairs and Communications and Ministry of Health, Labour and Welfare. During this period, 408 participants (13.1%) were lost to follow-up owing to their relocation. We censored them at the end of their relocation months. Serum carotenoid levels of participants in physical examinations were measured until the physical examination in 2011 (mean [SD] times for repeated measurements was 4.52 [4.71]).

**Figure.**
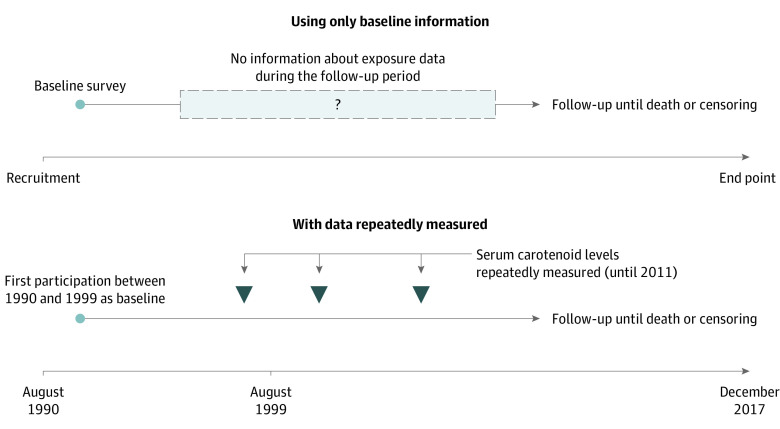
Study Design Using Repeated Measurements vs Baseline Measurements

This cohort study was conducted in accordance with the Declaration of Helsinki,^21^ and the study protocol was approved by the ethics review committee of Fujita Health University. Written informed consent was obtained from all participants. This study is reported following the Strengthening the Reporting of Observational Studies in Epidemiology (STROBE) reporting guideline.

### Exposure Assessment

We repeatedly measured serum carotenoid levels of eligible participants during the follow-up period. The samples were collected at the annual physical examinations and stored at −80 °C until carotenoid levels were measured in our laboratory. We assessed the serum levels annually of zeaxanthin and lutein, canthaxanthin, β-cryptoxanthin, lycopene, α-carotene, and β-carotene using high-performance liquid chromatography. The details of the measurement are described elsewhere.^22^ Based on high-performance liquid chromatography results, we calculated total carotene levels by summing α-carotene, β-carotene, and lycopene levels; total xanthophyll levels by summing zeaxanthin and lutein, canthaxanthin, and β-cryptoxanthin levels; provitamin A levels by summing β-cryptoxanthin, α-carotene, and β-carotene levels; and total carotenoid levels by summing the levels of all measured carotenoids.

### Outcome Assessment

After the baseline data collection from 1990 to 1999, municipal officials in Yakumo reviewed death certificates to assess the direct and indirect causes of death until December 2017. Based on this information, we assigned appropriate *International Classification of Diseases, Ninth Revision* (*ICD-9*) and *Tenth Revision* (*ICD-10*) codes for each death. The underlying causes of death were classified according to *ICD-9* for death certificates from 1990 to 1994 and *ICD-10* for death certificates from 1995 to December 2017 (ie, the end of the follow-up period). We transformed the codes from *ICD-9* into codes from *ICD-10* before our analysis. In this study, total mortality includes all *ICD-10* codes and cancer and CVD mortality were identified using the following *ICD-10* codes: C00-C96 (ie, malignant neoplasms) for cancer mortality and I00-I99 (ie, diseases of the circulatory system) for CVD mortality.

### Covariates

Eligible residents answered and completed the self-reported questionnaire, which included items regarding general health, lifestyle habits, medical history, clinical symptoms, and dietary habits before the health examination. In this study, lifestyle-related variables, such as tobacco smoking history (ie, current, ever, or never smoked) and alcohol consumption history (ie, current, ever, or never consumed alcohol), were collected. For each clinical condition, such as stroke, angina, diabetes, and cancer, participants were classified into 2 groups based on their answer in the questionnaire of never or yes (including ongoing treatment, past treatment, and no treatment for the condition). Fasting blood samples were collected during the physical examination and were centrifuged within 1 hour of sampling. Biochemical analyses for serum triglyceride and alanine aminotransferase (ALT) were conducted in Yakumo General Hospital laboratory. Systolic blood pressure (SBP) was measured using automatic upper-arm sphygmomanometers at the physical examination site after the participant had gotten at least 5 minutes of rest. Anthropometric measurements (ie, height and weight) were performed with help of trained public health nurses at the physical examination site. Body mass index (BMI) was calculated as weight in kilograms divided by height in meters squared.

### Statistical Analysis

Serum levels of triglyceride, ALT, and carotenoids had nongaussian distributions and therefore were expressed as medians and interquartile ranges (IQRs). These variables were used as log-transformed values in our statistical analysis. Other continuous variables with normal distributions were presented as means with SDs. The crude death rate was calculated according to quartiles and was presented as deaths per 1000 person-years. To estimate hazard ratios (HRs) and 95% CIs for the association of serum carotenoid levels with mortality using repeated measurements during follow-up, we performed a time-dependent Cox regression model.^23^ To ensure proportionality in this analysis, we performed the Schoenfeld method (eFigure 2 in the Supplement). The HRs in this analysis indicated a mortality risk in individuals for every 25% higher values in each carotenoid level. This meant, for example, mortality risk for every 14 μg/dL higher β-carotene levels (to convert to micromoles per liter, multiply by 0.01863) among the population and for every 5μg/dL higher β-cryptoxanthin levels among the population. For a detailed rationale for using the 25% difference in serum carotenoid levels, see the eAppendix in the Supplement. We included sex and age at baseline as potential confounders in the model adjusted for sex and age. Additionally, the fully adjusted model included sex, age, smoking habits, alcohol intake, SBP, triglyceride level, ALT level, and BMI. These covariates are also included as time-varying covariates in time-dependent Cox regression analyses. The rationale for inclusion as covariates in models was that these variables are associated with exposures (ie, carotenoids in this study) and outcomes (ie, mortality in this study).

As an additional analysis, we first compared the HR estimated in time-dependent Cox regression analysis with the HR using only the baseline levels of serum carotenoids. For analysis using the baseline data set, we applied Cox regression analysis, the common method in survival analysis. To examine different levels of increase in serum carotenoid levels, we performed statistical analyses with 4 different settings of serum carotenoid increase (ie, for every 15%, 20%, 25%, and 30% higher levels). To reduce potential bias from recent measurements, we also performed sensitivity analyses after the exclusion of individuals who measured serum carotenoids within the year before mortality and after stratification by sex.

Statistical significance was defined as a *P* value less than .05, and all tests were 2-sided. All statistical analyses were performed using R statistical software version 3.6.2 (R Project for Statistical Computing).

## Results

### Basic Characteristics and Recorded Death

Among 3116 individuals in the analysis, the mean (SD) age was 54.7 (10.6) years and 1883 (60.4%) were women; 2354 individuals survived (75.5%), and 762 individuals died (24.5%). The mean (SD) age was 63.2 (8.6) years for individuals who died and 52.0 (9.7) years for those who survived (Table 1). Among individuals who died, 432 (56.7%) were men, compared with 801 (34.0%) men among those who survived. During a median (IQR) follow-up of 22.3 (15.5-25.3) years, 762 deaths from all causes, 253 deaths from cancer, and 210 deaths from CVD were recorded.

**Table 1.** Baseline Characteristics of Participants by All-Cause Mortality

Characteristic	Median (IQR)	
	Alive (n = 2354)	Died (n = 762)
Age, mean (SD), y	52.0 (9.7)	63.2 (8.6)
BMI, mean (SD)	24.2 (3.3)	24.0 (3.3)
Triglycerides, mg/dL	100.0 (73.0-140.0)	113.5 (84.0-160.0)
ALT, IU/L	16.0 (11.0-23.0)	15.0 (11.0-22.0)
Systolic blood pressure, mean (SD), mm Hg	131.0 (20.1)	139.7 (21.1)
Zeaxanthin and lutein, μmol/L	1.09 (0.80-1.56)	1.09 (0.78-1.64)
Canthaxanthin, μmol/L	0.04 (0.03-0.06)	0.04 (0.02-0.06)
β-cryptoxanthin, μmol/L	0.31 (0.19-0.51)	0.27 (0.15-0.46)
Lycopene, mg/L	0.19 (0.11-0.32)	0.13 (0.78-0.22)
α-carotene, μmol/L	0.13 (0.09-0.18)	0.10 (0.07-0.15)
β-carotene, mg/L	48.1 (26.4-79.9)	35.9 (20.1-60.6)
Total carotene, μmol/L	1.45 (0.88-2.25)	1.09 (0.62-1.68)
Total xanthophylls, μmol/L	1.50 (1.11-2.15)	1.50 (1.04-2.15)
Provitamin A, μmol/L	1.42 (0.86-2.20)	1.09 (0.64-1.79)
Total carotenoid, mg/L	163.7 (114.3-237.3)	140.6 (96.6-209.3)
Men, No. (%)	801 (34.0)	432 (56.7)
Smoking history, No. (%)		
Never	1522 (64.7)	386 (50.7)
Ever	253 (10.7)	120 (15.7)
Current	579 (24.6)	256 (33.6)
Alcohol history, No. (%)		
Never	1330 (56.5)	397 (52.1)
Ever	54 (2.3)	47 (6.2)
Current	970 (41.2)	318 (41.7)
Clinical history, No. (%)		
Stroke	10 (0.4)	20 (2.6)
Angina	79 (3.4)	72 (9.4)
Diabetes	72 (3.1)	71 (9.3)

Abbreviations: ALT, alanine aminotransferase; BMI, body mass index (calculated as weight in kilograms divided by height in meters squared); IQR, interquartile range.

SI conversions factors: To convert ALT to microkatals per liter, multiply by 0.0167; β-carotene to micromoles per liter, multiply by 0.01863; carotenoid to micromoles per liter, multiply by 0.01863; lycopene to micromoles per liter, multiply by 1.863; triglycerides to millimoles per liter, multiply by 0.0113.

### All-Cause Mortality

In time-varying analyses adjusted for sex and age, individuals who had higher serum levels of most carotenoids in repeated measurements had statistically significantly lower risk for all-cause mortality (zeaxanthin and lutein: HR, 0.90; 95% CI, 0.87-0.93; *P* < .001; β-cryptoxanthin: HR, 0.90; 95% CI, 0.88-0.92; *P* < .001; lycopene: HR, 0.90; 95% CI, 0.88-0.92; *P* < .001; α-carotene: HR, 0.90; 95% CI, 0.88-0.92; *P* < .001; β-carotene: HR, 0.90; 95% CI, 0.89-0.92; *P* < .001; total carotene: HR, 0.88; 95% CI, 0.86-0.90; *P* < .001; total xanthophyll: HR, 0.87; 95% CI, 0.84-0.90; *P* < .001; provitamin A: HR, 0.88; 95% CI, 0.86-0.90; *P* < .001; and total carotenoid: HR, 0.84; 95% CI, 0.81-0.87; *P* < .001); however, individuals who had higher levels of canthaxanthin did not have lower risk of all-cause mortality (HR, 1.00; 95% CI, 0.98-1.03; *P* = 0.93) (Table 2). After further adjustment for time-varying smoking habits, alcohol intake, SBP, ALT levels, triglyceride levels, and BMI, higher serum canthaxanthin levels in repeated measurements were still not associated with a significantly lower risk for all-cause mortality (HR, 0.99; 95% CI, 0.97-1.02; *P* = .47). However, after adjustments, higher serum levels of other carotenoids in repeated measurements were associated with statistically significantly lower risk for all-cause mortality (zeaxanthin and lutein: HR, 0.90; 95% CI, 0.87-0.93; *P* < .001; β-cryptoxanthin: HR, 0.91; 95% CI, 0.88-0.93; *P* < .001; lycopene: HR, 0.91; 95% CI, 0.89-0.92; *P* < .001; α-carotene: HR, 0.90; 95% CI, 0.88-0.92; *P* < .001; β-carotene: HR, 0.91; 95% CI, 0.89-0.93; *P* < .001; total carotene: HR, 0.89; 95% CI, 0.87-0.91; *P* < .001; total xanthophyll: HR, 0.87; 95% CI, 0.84-0.90; *P* < .001; provitamin A: HR, 0.89; 95% CI, 0.87-0.91; *P* < .001; and total carotenoid: HR, 0.85; 95% CI, 0.82-0.87; *P* < .001).

**Table 2.** All-Cause Mortality by Repeated Measurement of Serum Carotenoid Levels

Carotenoid	Adjusted for sex and age		Fully adjusted^a^	
	HR (95% CI)^b^	*P* value	HR (95% CI)^b^	*P* value
Zeaxanthin and lutein	0.90 (0.87-0.93)	<.001	0.90 (0.87-0.93)	<.001
Canthaxanthin	1.00 (0.98-1.03)	.93	0.99 (0.97-1.02)	.47
β-cryptoxanthin	0.90 (0.88-0.92)	<.001	0.91 (0.88-0.93)	<.001
Lycopene	0.90 (0.88-0.92)	<.001	0.91 (0.89-0.92)	<.001
α-carotene	0.90 (0.88-0.92)	<.001	0.90 (0.88-0.92)	<.001
β-carotene	0.90 (0.89-0.92)	<.001	0.91 (0.89-0.93)	<.001
Total carotene	0.88 (0.86-0.90)	<.001	0.89 (0.87-0.91)	<.001
Total xanthophyll	0.87 (0.84-0.90)	<.001	0.87 (0.84-0.90)	<.001
Provitamin A	0.88 (0.86-0.90)	<.001	0.89 (0.87-0.91)	<.001
Total carotenoid	0.84 (0.81-0.87)	<.001	0.85 (0.82-0.87)	<.001

Abbreviation: HR, hazard ratio.

^a^ Adjusted for age, sex, smoking history, alcohol history, systolic blood pressure, alanine transaminase level, serum triglyceride level, and body mass index.

^b^ The HRs in this analysis indicate a mortality risk in individuals for every 25% higher value in a carotenoid level.

### Cancer Mortality

Similarly, higher levels of most serum carotenoids in repeated measurements were associated with statistically significantly lower cancer mortality risk in analyses adjusted for sex and age (zeaxanthin and lutein: HR, 0.89; 95% CI, 0.85-0.94; *P* < .001; β-cryptoxanthin: HR, 0.90; 95% CI, 0.86-0.93; *P* < .001; lycopene: HR, 0.90; 95% CI, 0.87-0.93; *P* < .001; α-carotene: HR, 0.87; 95% CI, 0.84-0.91; *P* < .001; β-carotene: HR, 0.89; 95% CI, 0.86-0.92; *P* < .001; total carotene: HR, 0.87; 95% CI, 0.83-0.90; *P* < .001; total xanthophyll: HR, 0.86; 95% CI, 0.81-0.91; *P* < .001; provitamin A: HR, 0.87; 95% CI, 0.84-0.91; *P* < .001; and total carotenoids: HR, 0.82; 95% CI, 0.78-0.86; *P* < .001) (Table 3). After multivariable adjustment for covariates, higher levels of serum carotenoids remained associated with statistically significantly lower risks of cancer mortality (zeaxanthin and lutein: HR, 0.89; 95% CI, 0.85-0.95; *P* < .001; β-cryptoxanthin: HR, 0.90; 95% CI, 0.86-0.94; *P* < .001; lycopene: HR, 0.90; 95% CI, 0.87-0.93; *P* < .001; α-carotene: HR, 0.87; 95% CI, 0.84-0.91; *P* < .001; β-carotene: HR, 0.90; 95% CI, 0.87-0.93; *P* < .001; total carotene: HR, 0.87; 95% CI, 0.84-0.90; *P* < .001; total xanthophyll: HR, 0.86; 95% CI, 0.81–-0.91; *P* < .001; provitamin A: HR, 0.88; 95% CI, 0.84-0.91; *P* < .001; and total carotenoid: HR, 0.82; 95% CI, 0.78-0.87; *P* < .001). However, higher levels of serum canthaxanthin in repeated measurements were not associated with statistically significantly lower cancer mortality risk in either model.

**Table 3.** Cancer Mortality by Repeated Measurements of Serum Carotenoid Levels

Carotenoid	Adjusted for sex and age		Fully adjusted^a^	
	HR (95% CI)^b^	*P* value	HR (95% CI)^b^	*P* value
Zeaxanthin and lutein	0.89 (0.85-0.94)	<.001	0.89 (0.85-0.95)	<.001
Canthaxanthin	0.96 (0.93-1.00)	.08	0.97 (0.93-1.01)	.17
β-cryptoxanthin	0.90 (0.86-0.93)	<.001	0.90 (0.86-0.94)	<.001
Lycopene	0.90 (0.87-0.93)	<.001	0.90 (0.87-0.93)	<.001
α-carotene	0.87 (0.84-0.91)	<.001	0.87 (0.84-0.91)	<.001
β-carotene	0.89 (0.86-0.92)	<.001	0.90 (0.87-0.93)	<.001
Total carotene	0.87 (0.83-0.90)	<.001	0.87 (0.84-0.90)	<.001
Total xanthophyll	0.86 (0.81-0.91)	<.001	0.86 (0.81-0.91)	<.001
Provitamin A	0.87 (0.84-0.91)	<.001	0.88 (0.84-0.91)	<.001
Total carotenoid	0.82 (0.78-0.86)	<.001	0.82 (0.78-0.87)	<.001

Abbreviation: HR, hazard ratio.

^a^ Adjusted for age, sex, smoking history, alcohol history, systolic blood pressure, alanine transaminase level, serum triglyceride level, and body mass index.

^b^ The HRs in this analysis indicate a mortality risk in individuals for every 25% higher value in a carotenoid level.

### CVD Mortality

In analyses adjusted for sex and age, higher levels of serum carotenoids, aside from canthaxanthin, in repeated measurements were associated with statistically significantly lower risk of CVD mortality (zeaxanthin and lutein: HR, 0.91; 95% CI, 0.86-0.97; *P* = 0.002; β-cryptoxanthin: HR, 0.91; 95% CI, 0.86-0.95; *P* < .001; lycopene: HR, 0.90; 95% CI, 0.87-0.93; *P* < .001; α-carotene: HR, 0.89; 95% CI, 0.85-0.93; *P* < .001; β-carotene: HR, 0.90; 95% CI, 0.87-0.93; *P* < .001; total carotene: HR, 0.88; 95% CI, 0.84-0.91; *P* < .001; total xanthophyll: HR, 0.88; 95% CI, 0.83-0.94; *P* < .001; provitamin A: HR, 0.88; 95% CI, 0.85-0.92; *P* < .001; and total carotenoid: HR, 0.84; 95% CI, 0.80-0.90; *P* < .001) (Table 4). In the fully adjusted analysis, higher levels of most serum carotenoids in repeated measurement were associated with statistically significantly lower risk for CVD mortality but with higher HRs (zeaxanthin and lutein: HR, 0.91; 95% CI, 0.86-0.97; *P* = .004; β-cryptoxanthin: HR, 0.92; 95% CI, 0.88-0.97; *P* < .001; lycopene: HR, 0.91; 95% CI, 0.87–0.94; *P* < .001; α-carotene: HR, 0.90; 95% CI, 0.86-0.94; *P* < .001; β-carotene: HR, 0.92; 95% CI, 0.88-0.96; *P* < .001; total carotene: HR, 0.89; 95% CI, 0.85-0.93; *P* < .001; total xanthophyll: HR, 0.89; 95% CI, 0.83-0.95; *P* < .001; provitamin A: HR, 0.90; 95% CI, 0.86-0.94; *P* < .001; and total carotenoid: HR, 0.86; 95% CI, 0.81-0.91; *P* < .001).

**Table 4.** Cardiovascular Disease Mortality by Repeated Measurements of Serum Carotenoid Levels

Carotenoid	Adjusted for sex and Age		Fully adjusted^a^	
	HR (95% CI)^b^	*P* value	HR (95% CI)^b^	*P* value
Zeaxanthin and lutein	0.91 (0.86-0.97)	.002	0.91 (0.86-0.97)	.004
Canthaxanthin	1.05 (1.00-1.10)	.048	1.04 (0.99-1.09)	.15
β-cryptoxanthin	0.91 (0.86-0.95)	<.001	0.92 (0.88-0.97)	<.001
Lycopene	0.90 (0.87-0.93)	<.001	0.91 (0.87-0.94)	<.001
α-carotene	0.89 (0.85-0.93)	<.001	0.90 (0.86-0.94)	<.001
β-carotene	0.90 (0.87-0.93)	<.001	0.92 (0.88-0.96)	<.001
Total carotene	0.88 (0.84-0.91)	<.001	0.89 (0.85-0.93)	<.001
Total xanthophyll	0.88 (0.83-0.94)	<.001	0.89 (0.83-0.95)	<.001
Provitamin A	0.88 (0.85-0.92)	<.001	0.90 (0.86-0.94)	<.001
Total carotenoid	0.84 (0.80-0.90)	<.001	0.86 (0.81-0.91)	<.001

Abbreviation: HR, hazard ratio.

^a^ Adjusted for age, sex, smoking history, alcohol history, systolic blood pressure, alanine transaminase levels, serum triglyceride levels, and body mass index.

^b^ The HRs in this analysis indicate a mortality risk in individuals for every 25% higher value in a carotenoid level.

### Comparison With Analysis Using Only Baseline Measurements

To evaluate the associations of carotenoid levels in analysis using repeated measurements with mortality risk, we compared the results from a time-dependent Cox regression model with the results using baseline serum carotenoid levels (eTable 1 in the Supplement). Compared with the analysis using baseline serum carotenoid levels, the decrease in risk for all-cause, cancer, and CVD mortality using repeated measurements was greater for most serum carotenoids; the HRs for all-cause, cancer, and CVD mortality were lower by 3% to 7%, 0% to 5%, and 4% to 10%, respectively. For example, the HR for higher levels of zeaxanthin and lutein using baseline measurements, compared with repeated measurements, was greater for all-cause mortality (0.95; 95% CI, 0.93-0.98; *P* = .003 vs 0.90, for a difference of 5.3%), cancer mortality (0.92; 95% CI, 0.87-0.97; *P* = .002 vs 0.89, for a difference of 3.3%), and CVD mortality (0.98; 95% CI, 0.93-1.04; *P* = .51 vs 0.91, for a difference of 7.1%). Overall, the HR was greater for higher total serum carotenoid levels using baseline measures, compared with repeated measurements, for all-cause mortality (0.92; 95% CI, 0.89-0.95; *P* < .001 vs 0.85, for a difference of 7.6%), cancer mortality (0.87; 95% CI, 0.83-0.93; *P* < .001 vs 0.82, for a difference of 5.7%), and CVD mortality (0.93; 95% CI, 0.88-0.99; *P* = .03 vs 0.86, for a difference of 7.5%).

### Sensitivity Analysis

As a sensitivity analysis, we examined the associations between serum carotenoid levels found via repeated measurements and all-cause, cancer, and CVD mortality with different ranges of increase in serum carotenoid levels (eTable 2, eTable 3, and eTable 4 in the Supplement). The results of the significance level tests remained identical, but the magnitude of the estimates changed according to differences in serum carotenoid levels. After excluding participants who had serum carotenoid levels measured within 1 year from death (eTable 5 in the Supplement), we found that the HRs for mortality of serum carotenoid levels found via repeated measurements were higher compared with the results in Table 2, Table 3, and Table 4. In a sex-stratified analysis, we found no difference between men and women in the associations between carotenoid levels and mortality risk, although serum levels in all carotenoids were significantly different by sex.

## Discussion

In this cohort study, we examined the association between carotenoid levels found via repeated measurements and mortality risk in a Japanese population. Over a follow-up period of 25 years, individuals with higher serum levels of most types of carotenoids had lower all-cause, cancer, and CVD mortality risk. We found that for results using repeated measurements, compared with those using baseline data only, most types of carotenoids had lower HRs for all-cause, cancer, and CVD mortality risk.

We examined the associations between serum carotenoid levels and all-cause and cause-specific mortality using a repeated measurement data set. An important limitation of previous studies was the use of single baseline measurements of carotenoid levels, which could not reflect changes in blood carotenoid levels during the follow-up period.^24,25^ We found that for results using repeated measurements, most carotenoids were associated with greater decreases in all-cause, cancer, and CVD mortality risk compared with results obtained using only baseline data. Despite a lack of a previous similar study, we speculated that our results may be associated with accumulation of antioxidant properties by continued high serum carotenoid levels during the follow-up period. Given that more recent measurements of an exposure variable are associated with risk of all-cause and cause-specific mortality, these decreased HRs in the time-dependent Cox regression model may be associated with the more recent measurements, which is referred to as the late-stage effect. Therefore, we addressed this issue and performed sensitivity analyses after excluding participants who had serum carotenoid levels measured within the past year before their deaths. However, we found no significant bias from the late-stage effect.

Previous cohort studies^7,8,9,10,11,24,25,26,27,28,29,30,31,32,33,34,35,36^ have examined the association between serum carotenoid levels and risk of all-cause, cancer, and CVD mortality. Most of these studies found inverse associations between serum carotenoid levels and mortality risk. Of these carotenoids, α-carotene, β-carotene, and lycopene are major antioxidants and play multiple bioactive roles in the human body.^37,38,39^ Moreover, a meta-analysis^40^ of prospective studies summarized the associations between the blood concentrations of different types of carotenoids and all-cause mortality, reporting that α-carotene, β-carotene, lycopene, and total carotene levels were inversely associated with mortality risk. As an example, for β-carotene, the authors reported that relative risk (RR) for each 25 μg/dL increase in serum level was 0.81 (95% CI, 0.72-0.90). We found a comparable association between serum β-carotene and all-cause mortality (HR for each 14 μg/dL increase in serum level, 0.91; 95% CI, 0.89-0.93). Although some interventional studies^34,41^ reported the null outcome or harmful cancer mortality outcomes associated with high-dose β-carotene supplementation, higher serum carotenoid levels did not have an association with increased risk of mortality in our study.

Serum carotenoid levels reflect an individual’s dietary fruit and vegetable intake.^42,43^ Based on the results of older and more recent studies, higher serum carotenoid levels may be attributed to the sufficient intake of fruits and vegetables, rather than high-dose supplementation of carotenoids, and these foods may include various micronutrients and be associated with beneficial outcomes. For β-cryptoxanthin, 3 prospective studies^7,24,32^ found an association between serum levels and mortality risk,^7,24,32^ and 1 study^40^ found an inverse association with mortality, with an HR of 0.85. In the current study, we also found a significant association between serum β-cryptoxanthin levels at baseline and all-cause mortality risk. Compared with the reported risk reduction of serum β-cryptoxanthin in mortality in Aune et al^40^ (RR for each 15 μg/dL increase in serum level, 0.84; 95% CI, 0.76-0.94), our study using repeated measurements found an association between β-cryptoxanthin levels and decreased all-cause mortality risk with an HR for each 5 μg/dL increase in serum level of 0.91 (95% CI, 0.88-0.93). The dietary intake of β-cryptoxanthin varies across countries worldwide, and Japan has one of the populations that commonly consumes this type of carotenoid.^44^ Therefore, blood β-cryptoxanthin levels have been found to be higher in Japanese populations than in European populations,^45^ which is likely to contribute to the associations between β-cryptoxanthin and CVD mortality as found in our study. Although 3 prospective studies^24,32,46^ examined the associations between zeaxanthin and lutein and mortality risk, the results were inconsistent. The study by Iribarren et al^47^ found an inverse association between zeaxanthin and lutein and atherosclerosis. Therefore, we hypothesized that serum zeaxanthin and lutein levels may be associated with decreased CVD mortality risk. Additionally, in our study, serum zeaxanthin and lutein levels at baseline were associated with lower all-cause and cancer-specific mortality risk. For canthaxanthin, there are no prospective studies on the association between blood levels and mortality. Our study found no association between serum canthaxanthin levels at baseline and all-cause or cause-specific mortality, whereas our study found that serum canthaxanthin levels are associated with some beneficial biological activities.^48,49^ The associations between zeaxanthin and lutein, β-cryptoxanthin, and canthaxanthin levels and mortality risk are still not fully elucidated, and further studies with long-term follow-up should be performed to validate these associations.

### Limitations

This study has several limitations. First, it was conducted among a homogenous population in Japan. Serum carotenoid levels found in this Japanese population were different from those of other ethnic populations. For example, the mean value of serum β-carotene in Japanese men from this study was 34.2 μg/dL, while the previously reported value for US men was 21.2 μg/dL.^31^ Therefore, these results may not be generalizable to other populations. Thus, further studies should be conducted to examine these associations in other, larger populations with repeated measurements of serum carotenoid levels. Second, unmeasured confounders may be associated with potential bias even in prospective studies. We tried to reduce the risk of bias by performing a sensitivity analysis and by adjusting for multiple covariates. However, owing to the limited items in our questionnaire in earlier periods, we could not adjust for important factors, such as physical activity and dietary quality. Third, as can happen in most studies using death certificates, nondifferential misclassifications for the cause of death could bias our findings toward the null (ie, bias the HR toward 1.00).^50^ Fourth, we did not incorporate the associations of multivitamin supplements with observed outcomes. Previous studies reported controversial results on the associations of multivitamin supplements with health outcomes.^51,52^ Therefore, although we cannot determine the direction of associations from dietary supplements in observed outcomes, it is highly possible that there was some kind of association. Fifth, in this study, 408 participants (13.1%) were lost to follow-up and censored. Although the percentage of loss to follow-up was relatively low, we cannot rule out biased estimation by loss to follow-up.^53^

## Conclusions

This study found that higher levels of serum carotenoids in an analysis using repeated measurements were associated with statistically significantly lower all-cause and cause-specific mortality risk over a follow-up period of 25 years, as reported in previous studies. In the time-dependent Cox regression analysis, we found that the decreases in mortality risk associated with lower serum levels of carotenoids were greater using repeated measurements compared with using baseline data alone. These results suggest that higher levels of serum carotenoids are associated with lower risk of all-cause, cancer, and CVD mortality.

## 

## Notes

Supplement. **eAppendix.** Rationale for Using 25% Difference in Serum Carotenoid Levels for Risk Estimation: Calculation Under Hypothetical Conditions **eFigure 1.** Flow Chart for Selection of Participants **eFigure 2.** Graphical Diagnostic for Proportionality Using Schoenfeld Method **eTable 1.** Comparison of HRs and 95% CIs for All-Cause and Cause-Specific Mortality by Baseline and Repeated Measurements **eTable 2.** Sensitivity Analysis of HRs and 95% CIs for All-Cause Mortality With Different Ranges of Increase **eTable 3.** Sensitivity Analysis of HRs and 95% CIs for Cancer Mortality With Different Ranges of Increase **eTable 4.** Sensitivity Analysis of HRs and 95% CIs for Cardiovascular Disease Mortality With Different Ranges of Increase **eTable 5.** HRs and 95% CIs for Mortality Risk Excluding Participants Who Measured Serum Carotenoid Levels Within 1 Year of Events
