# Supplementary material for: Analysis of Repeated Measurements of Serum Carotenoid Levels and All-Cause and Cause-Specific Mortality in Japan
Source: JAMA Netw Open. 2021 Jun 11;4(6):e2113369. doi: 10.1001/jamanetworkopen.2021.13369 (PMC8196342; doi:10.1001/jamanetworkopen.2021.13369)
Supplement: Supplement. — eAppendix. Rationale for Using 25% Difference in Serum Carotenoid Levels for Risk Estimation: Calculation Under Hypothetical Conditions eFigure 1. Flow Chart for Selection of Participants eFigure 2. Graphical Diagnostic for Proportionality Using Schoenfeld Method eTable 1. Comparison of HRs and 95% CIs for All-Cause and Cause-Specific Mortality by Baseline and Repeated Measurements eTable 2. Sensitivity Analysis of HRs and 95% CIs for All-Cause Mortality With Different Ranges of Increase eTable 3. Sensitivity Analysis of HRs and 95% CIs for Cancer Mortality With Different Ranges of Increase eTable 4. Sensitivity Analysis of HRs and 95% CIs for Cardiovascular Disease Mortality With Different Ranges of Increase eTable 5. HRs and 95% CIs for Mortality Risk Excluding Participants Who Measured Serum Carotenoid Levels Within 1 Year of Events [file jamanetwopen-e2113369-s001.pdf]

Supplemental Online Content

Fujii R, Tsuboi Y, Maeda K, Ishihara Y, Suzuki K. Analysis of repeated measurements of serum carotenoid levels and all-cause and cause-specific mortality in Japan. *JAMA Netw Open*. 2021;4(6):e2113369. doi:10.1001/jamanetworkopen.2021.13369

eAppendix. Rationale for Using 25% Difference in Serum Carotenoid Levels for Risk Estimation: Calculation Under Hypothetical Conditions
eFigure 1. Flow Chart for Selection of Participants
eFigure 2. Graphical Diagnostic for Proportionality Using Schoenfeld Method
eTable 1. Comparison of HRs and 95% CIs for All-Cause and Cause-Specific Mortality by Baseline and Repeated Measurements
eTable 2. Sensitivity Analysis of HRs and 95% CIs for All-Cause Mortality With Different Ranges of Increase
eTable 3. Sensitivity Analysis of HRs and 95% CIs for Cancer Mortality With Different Ranges of Increase
eTable 4. Sensitivity Analysis of HRs and 95% CIs for Cardiovascular Disease Mortality With Different Ranges of Increase
eTable 5. HRs and 95% CIs for Mortality Risk Excluding Participants Who Measured Serum Carotenoid Levels Within 1 Year of Events

This supplemental material has been provided by the authors to give readers additional information about their work.

© 2021 Fujii R et al. *JAMA Network Open.*

eAppendix. Rationale for Using 25% Difference in Serum Carotenoid Levels for Risk Estimation: Calculation Under Hypothetical Conditions

The reason why we decided “25% higher” for use in risk estimation is shown as follows. Let’s think beta carotene (MW = 536.87g/mol) as an example. In our sample, the median value of beta carotene was 0.834 µmol/L (536.87 * 0.834 = 447.75µg/L) in the baseline survey. Refer to this median value, the 25% higher level of beta carotene is 1.0425µmol/L (447.75 * 1.25 = 559.69µg/L). In other words, we estimated the hazard ratio compared with those who had 110µg/L higher (559.69 – 447.75 = 111.94 µg/L) in serum levels of beta carotenoid.

According to the Japanese food composition database, beta carotene is contained in 100g of raw (= 3/4 of a carrot), peel-less carrots at 7,200µg.[1] In addition, we need to consider bioavailability of beta carotene in human body. According to the previous study, 41.4 ± 7.4 % of the beta carotene was absorbed from raw, chopped carrot meals.[2] Taking information together, it is possible to absorb 2,981µg (7,200 * 0.414 = 2,980.8µg) beta carotene in our body when we eat 100g raw carrot. Furthermore, we assume that circulating blood volume in adults is 5L (5,000mL) in general. As a result, we can absorb 596.16µg/L (2,980.8 / 5 = 596.16µg/L) in our blood when we eat 100g raw carrot. 596.16µg/L seems to be much higher compared with 110µg/L.

---

[1] Ministry of Education, Culture, Sports, Science and Technology. Food composition database ver.2020. [Accessed on 29 March, https://fooddb.mext.go.jp/ in Japanese]
[2] Livny O, et al. β-carotene bioavailability from differently processed carrot meals in human ileostomy volunteers. Eur J Nutr. 2003; 42: 338-345.

© 2021 Fujii R et al. *JAMA Network Open.*

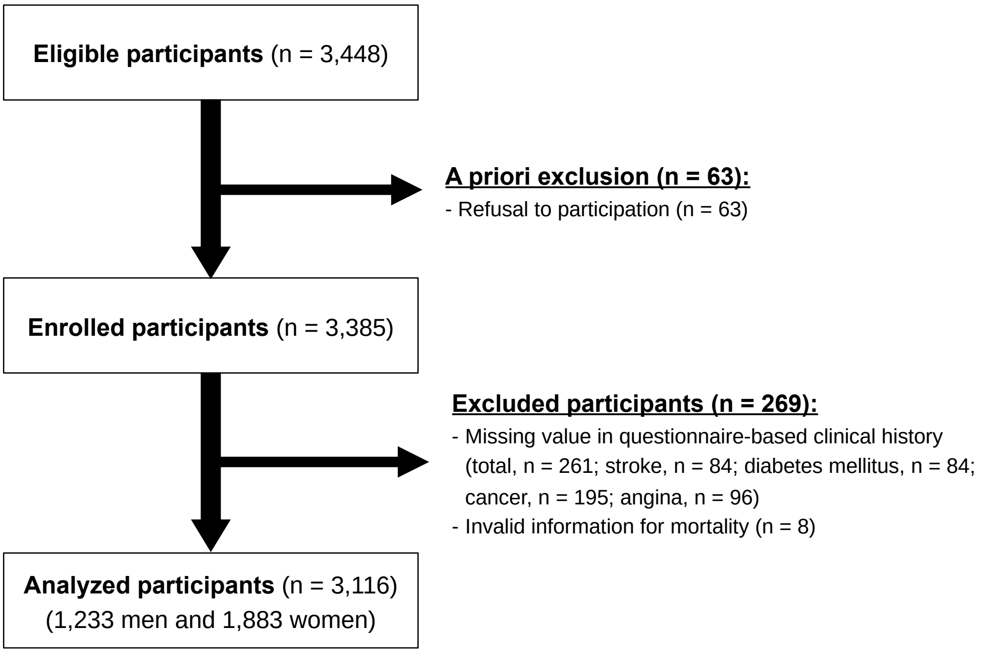

eFigure 1. Flow Chart for Selection of Participants

© 2021 Fujii R et al. *JAMA Network Open.*

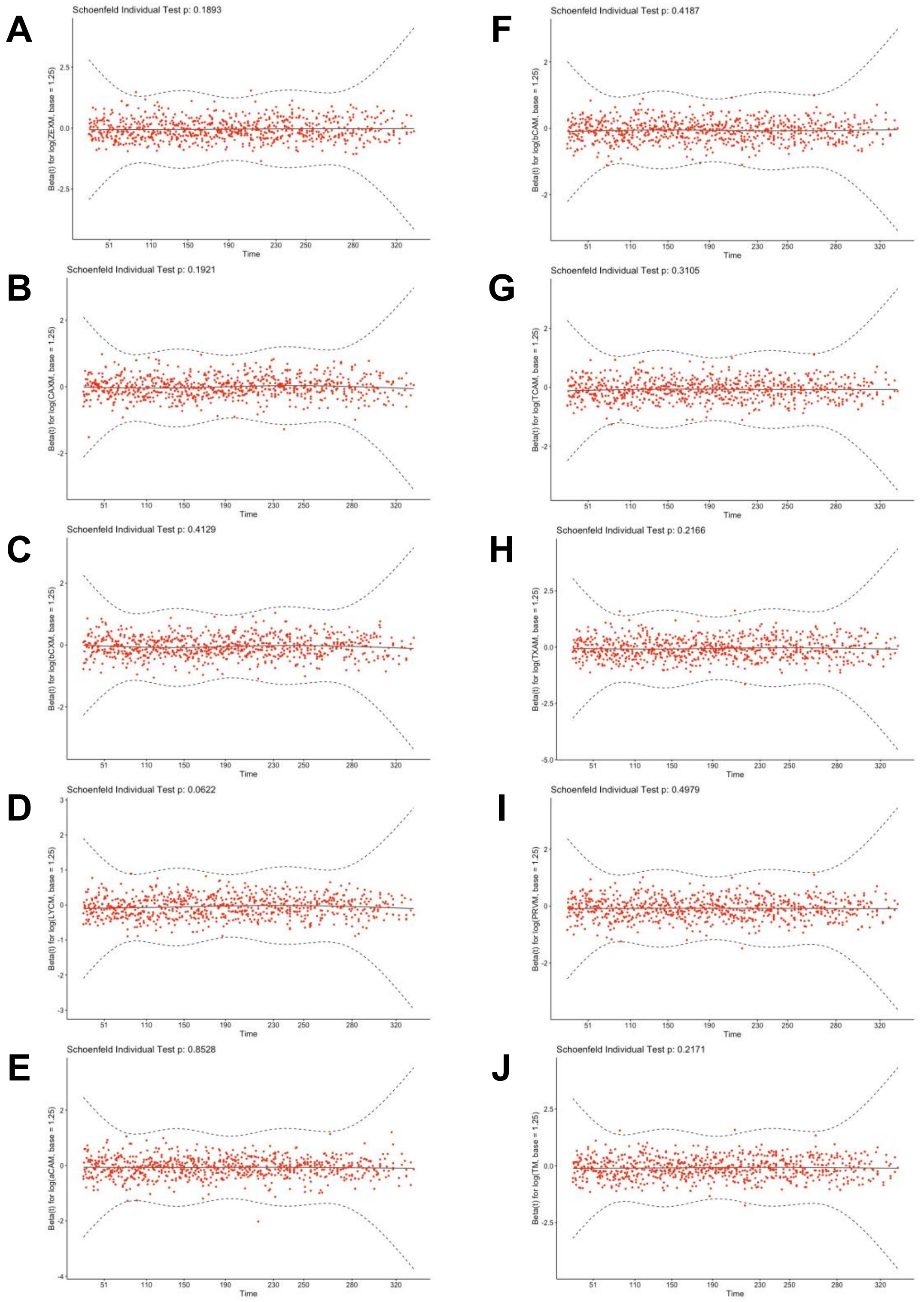

eFigure 2. Graphical Diagnostic for Proportionality Using Schoenfeld Method

A: Zeaxanthin/lutein; **B**: Canthaxanthin; **C**: β-cryptoxanthin; **D**: Lycopene; **E**: α-carotene; **F**: β-carotene; **G**: Total carotene; **H**: Total xanthophyll; **I**: Provitamin A; **J**: Total carotenoids.

© 2021 Fujii R et al. *JAMA Network Open.*

eTable 1. Comparison of HRs and 95% CIs for All-Cause and Cause-Specific Mortality by Baseline and Repeated Measurements[a]

	All-cause mortality				Cancer mortality				CVD mortality			
	Only baseline data		Repeated data		Only baseline data		Repeated data		Only baseline data		Repeated data	
	HR[b] (95% CI)	P-value	HR[b] (95% CI)	P-value	HR[b] (95% CI)	P-value	HR[b] (95% CI)	P-value	HR[b] (95% CI)	P-value	HR[b] (95% CI)	P-value
Zeaxanthin/lutein	0.95 (0.93–0.98)	0.003	0.90 (0.87–0.93)	<.001	0.92 (0.87–0.97)	0.002	0.89 (0.85–0.95)	<.001	0.98 (0.93–1.04)	0.51	0.91 (0.86–0.97)	0.004
Canthaxanthin	0.99 (0.97–1.02)	0.55	0.99 (0.97–1.02)	0.47	1.00 (0.96–1.04)	0.99	0.97 (0.93–1.01)	0.17	1.00 (0.96–1.05)	0.90	1.04 (0.99–1.09)	0.15
β-cryptoxanthin	0.95 (0.93–0.98)	<.001	0.91 (0.88–0.93)	<.001	0.96 (0.92–1.00)	0.036	0.90 (0.86–0.94)	<.001	0.97 (0.92–1.01)	0.16	0.92 (0.88–0.97)	<.001
Lycopene	0.95 (0.93–0.97)	<.001	0.91 (0.89–0.92)	<.001	0.93 (0.90–0.96)	<.001	0.90 (0.87–0.93)	<.001	0.95 (0.91–0.99)	0.017	0.91 (0.87–0.94)	<.001
α-carotene	0.93 (0.91–0.96)	<.001	0.90 (0.88–0.92)	<.001	0.91 (0.87–0.96)	<.001	0.87 (0.84–0.91)	<.001	0.92 (0.88–0.97)	0.002	0.90 (0.86–0.94)	<.001
β-carotene	0.94 (0.91–0.96)	<.001	0.91 (0.89–0.93)	<.001	0.91 (0.88–0.95)	<.001	0.90 (0.87–0.93)	<.001	0.93 (0.89–0.98)	0.002	0.92 (0.88–0.96)	<.001
Total carotene	0.93 (0.90–0.95)	<.001	0.89 (0.87–0.91)	<.001	0.90 (0.86–0.94)	<.001	0.87 (0.84–0.90)	<.001	0.93 (0.88–0.97)	0.002	0.89 (0.85–0.93)	<.001
Total xanthophyll	0.94 (0.91–0.98)	<.001	0.87 (0.84–0.90)	<.001	0.92 (0.87–0.97)	0.003	0.86 (0.81–0.91)	<.001	0.97 (0.92–1.04)	0.42	0.89 (0.83–0.95)	<.001
Provitamin A	0.92 (0.90–0.95)	<.001	0.89 (0.87–0.91)	<.001	0.90 (0.86–0.94)	<.001	0.88 (0.84–0.91)	<.001	0.93 (0.88–0.97)	0.003	0.90 (0.86–0.94)	<.001
Total carotenoid	0.92 (0.89–0.95)	<.001	0.85 (0.82–0.87)	<.001	0.87 (0.83–0.93)	<.001	0.82 (0.78–0.87)	<.001	0.93 (0.88–0.99)	0.028	0.86 (0.81–0.91)	<.001

CI, confidence interval; CVD, cardiovascular disease; HR, hazard ratio.

[a]Adjusted for age, sex, smoking habits, alcohol intake, systolic blood pressure, alanine transaminase levels, serum triglyceride levels, and body mass index.

[b]The HRs in this analysis indicate a mortality risk in those who had 25% higher values in each carotenoid level (μmol/L).

© 2021 Fujii R et al. *JAMA Network Open.*

eTable 2. Sensitivity Analysis of HRs and 95% CIs for All-Cause Mortality With Different Ranges of Increase[a]

	15% increase	20% increase	25% increase	30% increase
	**HR (95% CI)**	**HR (95% CI)**	**HR (95% CI)**	**HR (95% CI)**
Zeaxanthin/lutein	0.94 (0.92-0.96)	0.92 (0.89-0.94)	0.90 (0.87-0.93)	0.88 (0.85-0.92)
Canthaxanthin	0.99 (0.98-1.01)	0.99 (0.97-1.01)	0.99 (0.97-1.02)	0.99 (0.96-1.02)
β-cryptoxanthin	0.94 (0.93-0.95)	0.92 (0.90-0.94)	0.91 (0.88-0.93)	0.89 (0.86-0.92)
Lycopene	0.94 (0.93-0.95)	0.92 (0.91-0.94)	0.91 (0.89-0.92)	0.89 (0.87-0.91)
α-carotene	0.94 (0.92-0.95)	0.92 (0.90-0.94)	0.90 (0.88-0.92)	0.89 (0.86-0.91)
β-carotene	0.94 (0.93-0.96)	0.93 (0.91-0.94)	0.91 (0.89-0.93)	0.90 (0.87-0.92)
Total carotene	0.93 (0.91-0.94)	0.91 (0.89-0.92)	0.89 (0.87-0.91)	0.87 (0.84-0.89)
Total xanthophyll	0.92 (0.90-0.94)	0.89 (0.87-0.92)	0.87 (0.84-0.90)	0.85 (0.82-0.88)
Provitamin A	0.93 (0.92-0.95)	0.91 (0.89-0.93)	0.89 (0.87-0.91)	0.87 (0.85-0.90)
Total carotenoid	0.90 (0.88-0.92)	0.87 (0.85-0.89)	0.85 (0.82-0.87)	0.82 (0.79-0.85)

CI, confidence interval; HR, hazard ratio.

[a]Adjusted for age, sex, smoking habits, alcohol intake, systolic blood pressure, alanine transaminase levels, serum triglyceride levels, and body mass index.

© 2021 Fujii R et al. *JAMA Network Open.*

eTable 3. Sensitivity Analysis of HRs and 95% CIs for Cancer Mortality With Different Ranges of Increase[a]

	15% increase	20% increase	25% increase	30% increase
	HR (95% CI)	HR (95% CI)	HR (95% CI)	HR (95% CI)
Zeaxanthin/lutein	0.93 (0.90-0.97)	0.91 (0.87-0.96)	0.89 (0.85-0.95)	0.88 (0.82-0.94)
Canthaxanthin	0.98 (0.96-1.01)	0.98 (0.94-1.01)	0.97 (0.93-1.01)	0.97 (0.92-1.02)
β-cryptoxanthin	0.93 (0.91-0.96)	0.92 (0.88-0.95)	0.90 (0.86-0.94)	0.88 (0.84-0.93)
Lycopene	0.94 (0.92-0.96)	0.92 (0.89-0.95)	0.90 (0.87-0.93)	0.89 (0.85-0.92)
α-carotene	0.92 (0.90-0.94)	0.90 (0.87-0.93)	0.87 (0.84-0.91)	0.85 (0.82-0.90)
β-carotene	0.93 (0.91-0.95)	0.91 (0.89-0.94)	0.90 (0.87-0.93)	0.88 (0.84-0.91)
Total carotene	0.92 (0.89-0.94)	0.89 (0.86-0.92)	0.87 (0.84-0.90)	0.85 (0.81-0.89)
Total xanthophyll	0.91 (0.88-0.95)	0.89 (0.84-0.93)	0.86 (0.81-0.91)	0.84 (0.78-0.90)
Provitamin A	0.92 (0.90-0.94)	0.90 (0.87-0.93)	0.88 (0.84-0.91)	0.85 (0.82-0.90)
Total carotenoid	0.89 (0.86-0.92)	0.85 (0.82-0.89)	0.82 (0.78-0.87)	0.80 (0.75-0.85)

CI, confidence interval; HR, hazard ratio.

[a]Adjusted for age, sex, smoking habits, alcohol intake, systolic blood pressure, alanine transaminase levels, serum triglyceride levels, and body mass index.

© 2021 Fujii R et al. *JAMA Network Open.*

eTable 4. Sensitivity Analysis of HRs and 95% CIs for Cardiovascular Disease Mortality With Different Ranges of Increase[a]

	15% increase	20% increase	25% increase	30% increase
	HR (95% CI)	HR (95% CI)	HR (95% CI)	HR (95% CI)
Zeaxanthin/lutein	0.94 (0.91-0.98)	0.93 (0.88-0.98)	0.91 (0.86-0.97)	0.90 (0.84-0.97)
Canthaxanthin	1.02 (0.99-1.05)	1.03 (0.99-1.07)	1.04 (0.99-1.09)	1.04 (0.99-1.10)
β-cryptoxanthin	0.95 (0.92-0.98)	0.93 (0.90-0.97)	0.92 (0.88-0.97)	0.91 (0.86-0.96)
Lycopene	0.94 (0.92-0.96)	0.92 (0.90-0.95)	0.91 (0.87-0.94)	0.89 (0.85-0.93)
α-carotene	0.94 (0.91-0.96)	0.92 (0.88-0.95)	0.90 (0.86-0.94)	0.88 (0.84-0.93)
β-carotene	0.95 (0.93-0.97)	0.93 (0.90-0.96)	0.92 (0.88-0.96)	0.91 (0.86-0.95)
Total carotene	0.93 (0.91-0.96)	0.91 (0.88-0.94)	0.89 (0.85-0.93)	0.87 (0.83-0.92)
Total xanthophyll	0.93 (0.89-0.97)	0.91 (0.86-0.96)	0.89 (0.83-0.95)	0.87 (0.80-0.94)
Provitamin A	0.94 (0.91-0.96)	0.92 (0.89-0.95)	0.90 (0.86-0.94)	0.89 (0.84-0.93)
Total carotenoid	0.91 (0.87-0.94)	0.88 (0.84-0.93)	0.86 (0.81-0.91)	0.83 (0.78-0.90)

CI, confidence interval; HR, hazard ratio.

[a]Adjusted for age, sex, smoking habits, alcohol intake, systolic blood pressure, alanine transaminase levels, serum triglyceride levels, and body mass index.

© 2021 Fujii R et al. *JAMA Network Open.*

eTable 5. HRs and 95% CIs for Mortality Risk Excluding Participants Who Measured Serum Carotenoid Levels Within 1 Year of Events[a]

	All-cause mortality		Cancer mortality		CVD mortality	
	HR[b] (95% CI)	P-value	HR[b] (95% CI)	P-value	HR[b] (95% CI)	P-value
Zeaxanthin/lutein	0.95 (0.93–0.98)	0.003	0.90 (0.87–0.93)	<.001	0.92 (0.87–0.97)	0.002
Canthaxanthin	0.99 (0.97–1.02)	0.55	0.99 (0.97–1.02)	0.47	1.00 (0.96–1.04)	0.99
β-cryptoxanthin	0.95 (0.93–0.98)	<.001	0.91 (0.88–0.93)	<.001	0.96 (0.92–1.00)	0.036
Lycopene	0.95 (0.93–0.97)	<.001	0.91 (0.89–0.92)	<.001	0.93 (0.90–0.96)	<.001
α-carotene	0.93 (0.91–0.96)	<.001	0.90 (0.88–0.92)	<.001	0.91 (0.87–0.96)	<.001
β-carotene	0.94 (0.91–0.96)	<.001	0.91 (0.89–0.93)	<.001	0.91 (0.88–0.95)	<.001
Total carotene	0.93 (0.90–0.95)	<.001	0.89 (0.87–0.91)	<.001	0.90 (0.86–0.94)	<.001
Total xanthophyll	0.94 (0.91–0.98)	<.001	0.87 (0.84–0.90)	<.001	0.92 (0.87–0.97)	0.003
Provitamin A	0.92 (0.90–0.95)	<.001	0.89 (0.87–0.91)	<.001	0.90 (0.86–0.94)	<.001
Total carotenoid	0.92 (0.89–0.95)	<.001	0.85 (0.82–0.87)	<.001	0.87 (0.83–0.93)	<.001

CI, confidence interval; CVD, cardiovascular disease; HR, hazard ratio.

[a]Adjusted for age, sex, smoking habits, alcohol intake, systolic blood pressure, alanine transaminase levels, serum triglyceride levels, and body mass index.

[b]The HRs in this analysis indicate a mortality risk in those who had 25% higher values in each carotenoid level (μmol/L).

© 2021 Fujii R et al. *JAMA Network Open.*
